# The cellulose fibers functionalized with star-like zinc oxide nanoparticles with boosted antibacterial performance for hygienic products

**DOI:** 10.1038/s41598-022-05458-7

**Published:** 2022-01-25

**Authors:** M. Onyszko, A. Markowska-Szczupak, R. Rakoczy, O. Paszkiewicz, J. Janusz, A. Gorgon-Kuza, K. Wenelska, E. Mijowska

**Affiliations:** 1 Department of Nanomaterials Physicochemistry, Faculty of Chemical Technology and Engineering, West Pomeranian University of Technology, Szczecin, Piastow Ave. 42, 71-065 Szczecin, Poland; 2 Department of Chemical and Process Engineering, Faculty of Chemical Technology and Engineering, West Pomeranian University of Technology, Szczecin, Piastow Ave. 42, 71-065 Szczecin, Poland; 3 Arctic Paper Kostrzyn SA, ul. Fabryczna 1, 66-470 Kostrzyn nad Odra, Poland

**Keywords:** Microbiology, Materials science

## Abstract

Bacterial infectious diseases are serious health problem which extends to economic and social complications. Moreover, bacterial antibiotic resistance, lack of suitable vaccine or emergence of new mutations is forcing the development of novel antimicrobial agents. The objective of this study is to synthesize and characterize star-like zinc oxide nanoparticles for the application of antibacterial activities in cellulose based hygiene products. ZnO NPs were in situ synthesized via precipitation method on the surface of cellulose fibers. Since bactericidal activity of nanoparticles in part depends on the concentration in the growth medium, various amount of ZnO was incorporated into cellulose matrix ranging from 1 to 3 wt%. Microscopic (TEM, SEM) and spectroscopic (FT-IR, XRD) methods were utilized to investigate the final products. The infrared absorption spectra analysis supported by theoretical finding that during the reaction, ZnO nanoparticles could be bonded with cellulose fibers via hydrogen bonding. The yield of functionalization was determined through thermogravimetric analysis. Collected data proved the successful functionalization of the cellulose fibers with nanoparticles. Static contact angle measurements were carried out showing absorptive character of as prepared fabrics. All the samples were tested for the antibacterial properties and the results were compared to the samples prepared from the pristine cellulose fibers. Moreover, mechanical tests were performed revealing that the addition of only 2 wt% of the nanofiller boosted tensile, tearing and bursting strength by a factor of 1.6, 1.4 and 2.2 in comparison to unfunctionalized paper sample, respectively. Fabricated fabric presenting high hydrophilicity and antibacterial properties have gained increased applications in fabric industry, including hygiene product industry and hence the result of this study would be a welcomed option.

## Introduction

Rapid growth of medical science in the twenty-first century has brought a number of discoveries and advances, giving people the chance to live a long life. Over the years mankind has learnt how to defeat infectious organisms or at least inhibit or prevent the growth of microorganisms. Nevertheless, it is impossible to change the fact that bacteria can adjust to the constant fluctuations in their environment and become resistant to antibiotics (Antibiotic Resistant Bacteria, ABR), drugs and other chemical agents which dramatically suppresses their effectiveness. Thus it is essential to investigate and systematically provide new antibacterial agents in order to avoid the development of serious diseases. In this case nanotechnology can be very useful tool. It is one of the fastest growing section of applied science and engineering that have been introduced to the world in this century. It covers the characterization and preparation of devices and materials that have at least one dimension that is about 1–100 nm. Nowadays, not only developed but also developing countries are investing in this fascinating and promising technology^1^. It utilizes the beneficial properties of nanosized materials in comparison to their bulk counterparts which are attributed to the small sizes, high surface area and large amount of surface atoms and surface active sites^2^. This phenomenon involves enhancement of such aspects as: mechanical strength, chemical reactivity^3^, optical, electric and magnetic properties^4^. Current advances in nanotechnology give the possibility to produce nanomaterials with specific sizes and shapes. Therefore, tailoring important properties of nanosized materials has become possible. All of this resulted in a wide application potential of nanomaterials ranging from food and cosmetics industry to medicine, environmental health or automotive and construction industry^5^.

Recently, nanotechnology has started to play a major role in the development of antibacterial agents. Many microorganisms, for example bacteria, exist in the range from hundreds of nanometers to tens of micrometers. Their outer cellular membrane contains nanometer-sized pores where crossing of the cell membrane is the most likely to happen. It is understandable that only particles of the same or smaller size can successfully pass through these pores and consequently exhibit distinct bactericidal mechanism^6^. Thus, small size of materials with antibacterial function is particularly important. Among the number of nanomaterials currently used in the field of nanobiotechnology metal and metal oxides nanoparticles are considered to be the most promising ones. Among the inorganic oxides, titanium dioxide (TiO_2_), zinc oxide (ZnO), magnesium oxide (MgO) or copper oxide (CuO) have been widely used as an antimicrobial agent due to their good stability and safety towards humans and animals^7–10^.

In a view of textile industry development, it is no wonder that nanotechnology has found its way to this sector very quickly. The novel properties and low amount of material consumption are the main reasons for such vast interest in nanotechnology application. Textile and fibers surface modification provides a way to impart new and desirable properties to the fabric while retaining comfort and maintain or even enhance mechanical strength. Health concerns and customer satisfaction drive fast-paced growth of functional textiles industry. In case of functional finishes that alter fiber or fabric performance, are applied specifically modification to amend properties related to care, comfort and durability. Most functional fabric properties can be imparted with the use of various nanomaterials^11^.

Recently, for example, many studies have been devoted to the preparation of cellulose as well as its modification and application. Natural fiber products are highly popular these days and widely applied in daily life. It is because of their superb properties such as biodegradability, softness and absorbing nature. When it comes to cellulose fibers the ease of functionalization and modification due to the abundance of OH functional groups is also an important factor that makes this material desirable for many applications. However, it provides convenient substrate to grow microorganisms especially at appropriate temperature and humidity level. Therefore, numerous chemicals have been utilized to enriched cellulose fibers with antibacterial features.

Among nano-sized antibacterial agents, silver nanoparticles (AgNPs) are widely used. However, the shortcomings of textile yellowing along with high production costs AgNPs are the main reasons for the limitation of their applications. Moreover, possible development of resistance due to the wide usage of AgNPs results in the searching for new antimicrobial nanomaterials. In regard to CuO nanoparticles the main drawback for their use is due to potentially toxic effect. Karlsson et al. reported that CuO NPs showed relatively high toxicity to human lung cultured cells and for human skin organ cultures^12^. Titanium dioxide nanoparticles are also highly considered by researchers because of their antibacterial properties and low-toxicity. But there is a need to apply light irradiation in the UV range what additionally limits its efficiency^13^.

On the contrary ZnO nanomaterials, especially ZnO nanoparticles (ZnO NPs) have recently attracted much researcher’s attention^14,15^. It possess some significant advantages that make it one of the most future-oriented metal oxide antimicrobial agent. Its non-toxicity towards human cells is a highly important factor that allows its usage in components having contact with human tissues and cells^16^ or with food^17^. Relatively low cost and variety of synthesis methods are crucial benefits from the economical point of view, allowing its large scale production and utilization. Additionally, good thermal stability as well as surface activity contribute to the ease of its processing. Thanks to the large surface-to-volume ratio of the ZnO nanoparticles, the antimicrobial performance of this material may reach high values. Moreover, there is no requirement for using the light irradiation to achieve and exploit its antibacterial activity and only small amount of nanoparticles is regarded to obtain sufficient degree of the bacterial growth inhibition.

Elemike et al. have prepared crystalline cellulose–ZnO nanoparticles composite using corn cobs to isolate cellulose. The nanocomposite displayed improved antibacterial action especially toward *Escherichia coli*^18^*.* Gao et al. have also prepared cellulose and cellulose composites membranes using TiO_2_, chitosan as well as ZnO nanoparticles. All the composite membranes demonstrated strong antibacterial activity against both *Staphylococcus aureus* and *E. coli* due to dispersion of antibacterial agents in the composite membranes via phase inversion method^19^. In another study Ali and coworkers have impregnated cellulose isolated from citrus peel waste with Zinc oxide nanoparticles. The zinc cellulose nanocomposite paper discs have shown better antibacterial activity in comparison to pure cellulose and ZnO nanoparticles^20^.

In this study, star-like ZnO nanoparticles were in situ synthesized and simultaneously incorporated into cellulose matrix via precipitation method to evaluate the antibacterial activity of obtained nano-composite. Obtained material was characterized by FT-IR, XRD, TGA and its morphology examination was developed by TEM and SEM methods. As prepared, functionalized fibers were utilized to prepare cellulose pads. Samples containing 1 wt%, 2 wt%, and 3 wt% of nanomaterial were produced and tested for their antibacterial activity. All composite materials exhibited interesting properties against both Gram-negative and Gram-positive model bacteria. Furthermore, their mechanical properties have been also described. Moreover, the surface wettability of untreated and treated cellulose fabrics was investigated by measuring the contact angle of a water droplet revealing that the incorporation of ZnO nanoparticles has not significantly changed its profile. The impregnated fabric can still act as a water absorbing material making it useful for such application as antibacterial hygiene products or textiles.

## Experimental

### Materials

Zinc acetate dihydrate (Zn(CH_3_COO)_2_ × 2H_2_O) was purchased from Sigma Aldrich (Poland). Sodium hydroxide (NaOH) was supplied from Chempur (Poland). Bleached craft pulps, consisted of short eucalyptus cellulose fibers and long wood cellulose fibers (4 g/dm^3^), were obtained from Arctic Paper Kostrzyn S.A. Poland.

### In situ functionalization of cellulose fibers

To prepare functionalized cellulose fibers, 17 mL of cellulose pulp containing 680 mg of short cellulose fibers and 1.75 mL of cellulose pulp containing 70 mg of long cellulose fibers (9:1 *w*/*w*) were diluted in 250 mL of distilled water. The mixture was stirred with a magnetic stirrer at 300 rpm for 20 min to obtain homogenous slurry. The impregnation of ZnO nanoparticles was performed with modified reported precipitation method^21^. First, the desired amount of Zn(CH_3_COO)_2_ × 2H_2_O was dissolved in the cellulose slurry and the mixture was stirred for an hour. Then, in order to obtain ZnO nanoparticles on the surface of cellulose fibers, the appropriate volume of 0.01 M NaOH solution corresponding to 100 mg of NaOH per 46 mg of zinc acetate dihydrate was added dropwise using the syringe pump (Programmable Microfluidics Syringe Pump, NE-1002X) with the infusion rate of 1 mL/min. Subsequently, the cellulose slurry was washed with ethanol and obtained residue was filtrated and washed with distilled water until the pH value approached 7. Modified cellulose fibers were then immersed in 250 mL of distilled water. All the procedures were performed in an ambient conditions.

The yield of ZnO nanoparticles formation in cellulose matrix C/ZnO (cellulose modified with ZnO nanoparticles) was determined experimentally and it was ca. 100%. The amounts of individual chemicals and materials used for the preparation of nanocomposites are listed in the Table 1 below.

**Table 1:** Chemical and materials used for the preparation of nanocomposites.

Name of the sample	Chemical and materials used for the preparation of nanocomposites
C/ZnO 1%	680 mg of short cellulose fibers
	70 mg of long cellulose fibers
	20.27 mg of Zn(CH_3_COO)_2_ × 2H_2_O
	125 mL of 0.01 M NaOH
C/ZnO 2%	680 mg of short cellulose fibers
	70 mg of long cellulose fibers
	40.54 mg of Zn(CH_3_COO)_2_ × 2H_2_O
	250 mL of 0.01 M NaOH
C/ZnO 3%	680 mg of short cellulose fibers
	70 mg of long cellulose fibers
	60.81 mg of Zn(CH_3_COO)_2_ × 2H_2_O
	375 mL of 0.01 M NaOH

### Fabrication of the functionalized cellulose pads

The cellulose pads were prepared using the filtration set (filtration set all glass filter holder with NS and sintered disc-set). Each sample was obtained from 18 mL of as prepared functionalized cellulose pulp. The volume of the pulp was determined experimentally and allowed to produce pads weighted ~ 0.1 g with good reproducibility. Modified cellulose paper pads containing 1 wt%, 2 wt%, and 3 wt% of nanofiller per 1 g of dry cellulose fibers were fabricated. The control samples were prepared in the same manner but without the nanofiller. Before the antibacterial tests were conducted the samples were dried and stored at ambient conditions.

## Characterization

The morphology of the obtained materials was examined via transmission electron microscopy (TEM, Tecnai F30) and scanning electron microscopy (SEM, VEGA3 TESCAN). X-ray diffraction (XRD) patterns were carried out using X’Pert Philips Diffractometer with Cu lamp (Kα1 = 1.54056 Å) to investigate the crystal composition of the samples. Fourier transform infrared spectroscopy (FT-IR) was used to determine the functional groups on the surface of cellulose fibers. The absorption spectra were recorded on Nicolet 6700 FT-IR Spectrometer. For the measurements the cellulose pads samples were grinded using ball mill (agate grinding balls, 10 mm diameter, 500 rpm, 15 min) and the ZnO powder was synthesized via precipitation method but without the presence of cellulose. Thermogravimetric analysis (TGA) was carried out on 10 mg samples using a DTA-Q600 SDT TA at a heating rate of 10 °C/min from room temperature to 550 °C under air. Static contact angle measurements were carried out by the sessile drop method using a Krüss DSA4 apparatus. An average of five measurements with water was made to determine the wettability of the surfaces. The volume of the individual droplet used for the static contact angle measurements was 5 μL. To calculate the contact angle ImageJ program was used.

### Antibacterial test

The antimicrobial test of properties of functionalized cellulose paper pads and control sample were conducted for Gram-negative bacteria *E. coli* K12 (ATCC 29425) and Gram-positive bacteria *Staphyloccocus epidermidis* (ATCC 49461). The test microorganisms were cultivated in nutrient broth (NB, BioMaxima Poland) for *E. coli* and Brain–Heart Infusion (BHI, BioMaxima Poland) for *S. epidermidis* and incubated for 24 h at 37 °C. The bacterial cells were centrifuged at 5000 g at 25 °C for 10 min and resuspended in 0.85% sodium saline buffer. The bacterial cultures were adjusted to the final concentration 0.5 in McFarland standard to give a final concentration approx. 1 × 10^8^ CFU/mL (working bacterial suspension). The experiments were prepared according to Zemjicet al.^22^ with some modifications. For the antibacterial performance examination the samples of functionalized cellulose pads or raw cellulose pad (control) were cut into circles (Ø = 35 ± 1 mm) and transferred into 250 mL bottles containing 55 mL of working bacterial suspension. The suspension was continuously agitated using a magnetic stirrer (120 rpm). Samples were collected after 3 h and 24 h. The collected bacterial solution were diluted with sodium saline buffer by a factor from 10^1^ to 10^4^. Next, we plated 250 μL of diluted nutrient broth onto standard Standard Plate Count Agar (PCA) for *E. coli* and Brain–Heart Infusion Agar (BHI) for *S. epidermidis*. The inculcated plates were incubated at a temperature of 37 °C for 24 h. Then the visible colonies were counted and shown as log CFU/mL. The antimicrobial activity was expressed as bacterial survival rate after contact with the functionalized paper and control sample. All the experiments were performed in triplicate and data were analyzed by a Student’s *t*-test and a value of *p* < 0.05 was considered significant. Analyzes were performed using Microsoft excel.

### Luminometric analyses

ATP bioluminescence measurements were performed using the LuciPac A3 Surface swabs and Lumitester Smart Kikkoman Biochemifa (Japan) reader. The measurements were carried out to derive threshold values of cellulose fibers and fibers functionalized with 3 wt% of ZnO nanoparticles. Than cellulose pads were immersed in bacterial suspension with the final concentration of 0.5 in McFarland (approx. 1 × 10^8^ CFU/mL). ATP bioluminescence reaction was assumed after pulling and after 1.5 h incubation at temperature of 37 °C. The results of the ATP measurements were expressed as Relative Light Units (RLU).

### Mechanical properties

Mechanical properties of functionalized and unfunctionalized samples were investigated. Here, the model paper samples were manufactured under pilot conditions at the Arctic Paper Kostrzyn S.A. Poland on a Rapid-Kothen. 20 cm in diameter paper discs were prepared. The paper samples were made without the use of sizing and bleaching substances. Therefore, model paper samples were pristine short and long cellulose fibers. The mechanical tests were carried out at the Arctic Paper company in air-conditioning room (23 °C, 50% of the humidity level) according to ISO 5270 standard. Tearing resistance was determined using an Elmendorf apparatus (Lorentzen & Wettre, Zurich, Switzerland) according to ISO 1924-2. The width and length of the tested paper strips were 15 and 100 mm, accordingly. The tensile strength measurements were conducted on the automatic tensile tester (Messmer Büchel, K465, Veenendaal, The Netherlands), according to ISO 1974 on samples consisting of 4 paper sheets. The bursting strength was tested on the bursting strength tester (Messmer Büchel, Veenendaal, The Netherlands) according to ISO 2758 on paper samples with dimensions of more than 70 mm × 70 mm.

## Results and discussion

**Figure 1:**
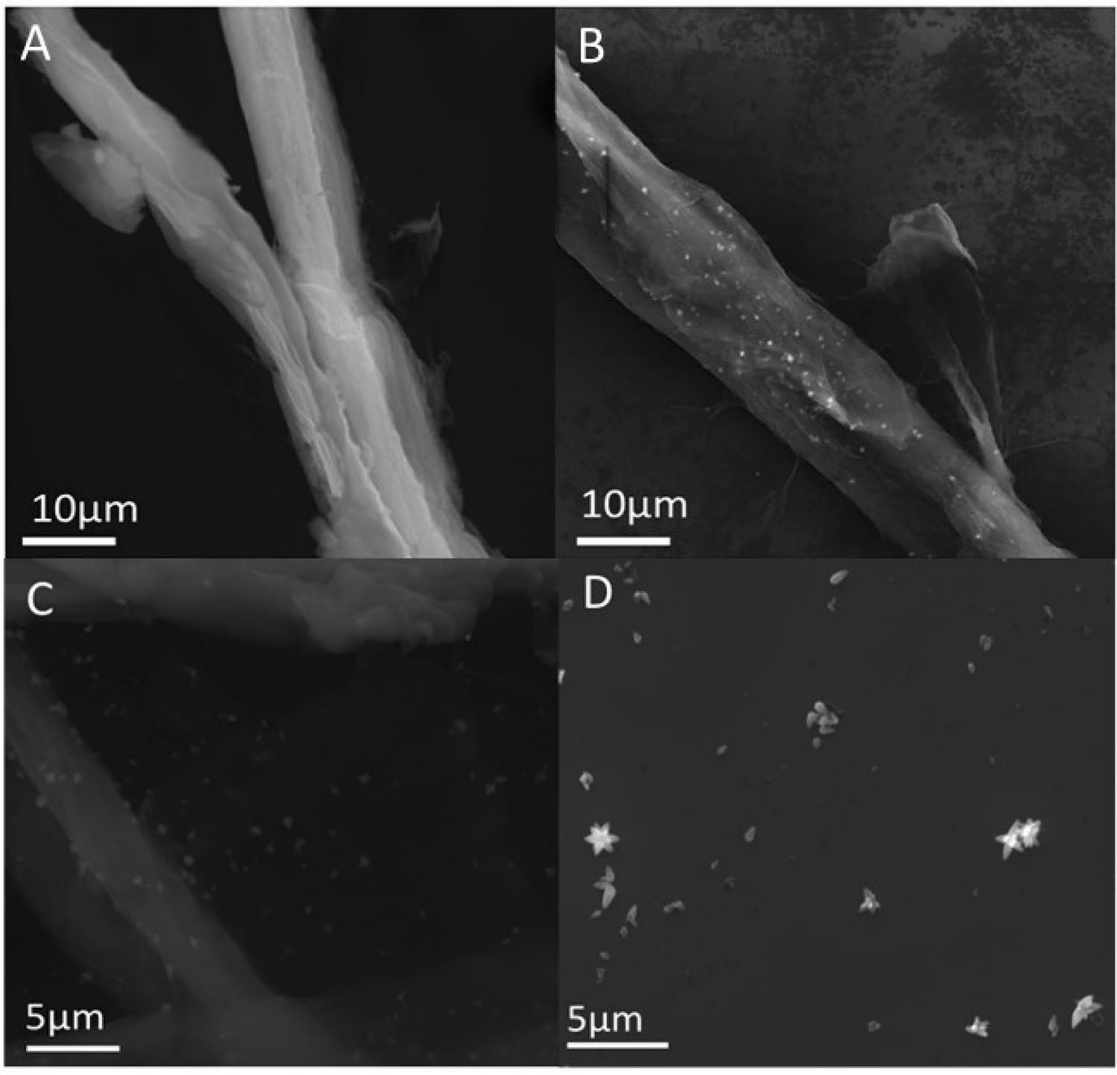
SEM images of pure cellulose fibers (**A**) and cellulose fibers functionalized with ZnO nanoparticles (**B**, **C**), ZnO nanoparticles after ball-milling of the composite (**D**).

The morphology of cellulose fibers before and after the functionalization was characterized using scanning electron microscope (Fig. 1A–D). Figure 1A presents the typical hierarchical structure of bleached softwood cellulose fibers. The surface of the unmodified cellulose is smooth and macrofibrils, that run in the direction of the fiber, are observed. Figure 1B depicts cellulose fibers after the functionalization. The formation of the ZnO nanoparticles on the surface of fibers is revealed. The precipitation method resulted in the formation of star-shaped structures with the average size of individual nanoparticle < 2 µm. 3D morphology of the obtained nanoparticles were uniformly distributed on the cellulose fibers. In the lower magnification image (Fig. 1B, C) no clusters were detected, which indicates that slow process of NaOH addition prevented the agglomeration of ZnO nanoparticles. For better nanoparticles visualization the synthesized ZnO on cellulose fibres the composite was ball-milled. Subsequently, SEM analysis of the obtained sample was conducted and presented in Fig. 1D showing clear star-like morphology (some of them were destroyed during ball milling).

**Figure 2:**
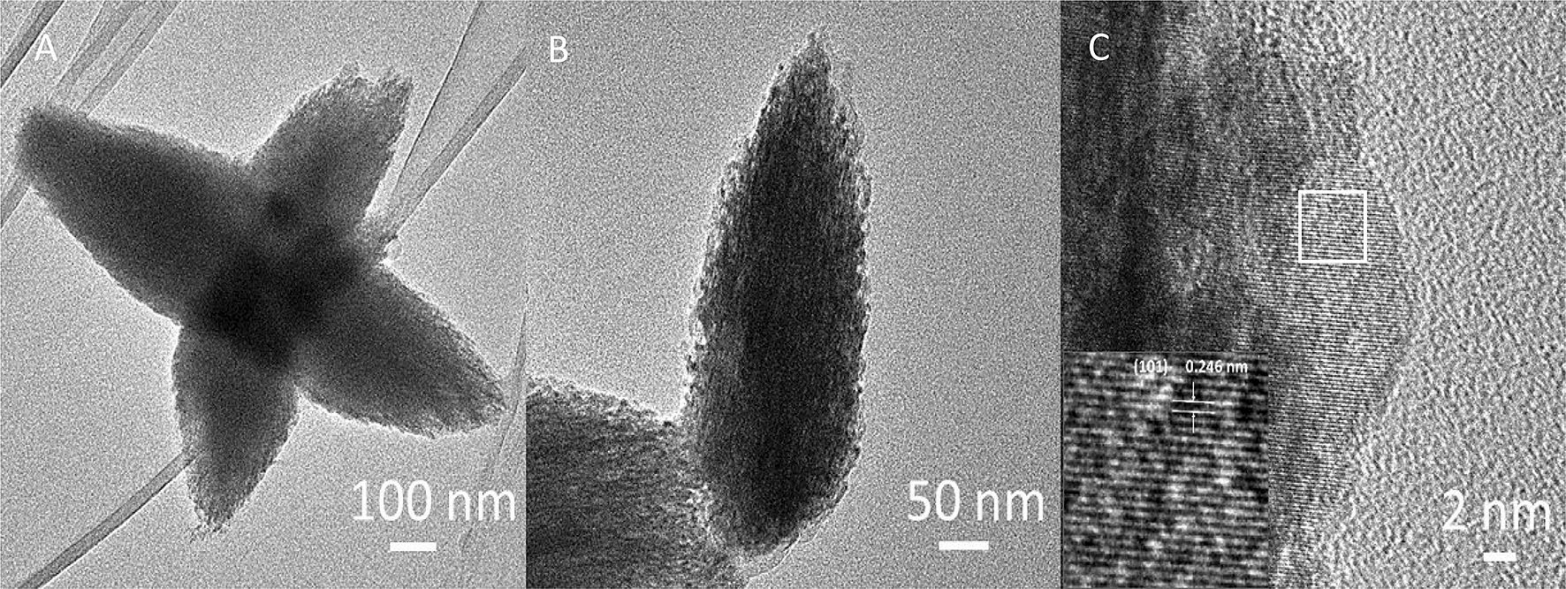
TEM images of ZnO nanoparticles with different magnifications (**A**–**C**).

For the further investigation of the morphology of the obtained ZnO NPs TEM analysis was performed (Fig. 2A–C). In Fig. 2A star-like structures are presented. Each particle is composed of uniform cones growing from the center. Magnified image shows that star-shaped structures are constituted by the accumulation of several sharp-tipped trigonal discs. Figure 2B displays a single cone with a pyramid tip. The typical width and length of an individual cone are in the range of 0.550 µm ± 0.12 µm and 0.243 µm ± 0.037 µm, respectively. The inset in Fig. 2C shows high resolution image. The lattice plane in ZnO was identified and the experimental value obtained for d_(101)_ ≈ 0.246 nm was very close to the theoretically predicted (0.248 nm). Other approach to fabricate star-like ZnO structures are presented by Ghayempour and Montazer. They obtained irregular ZnO star- like nanoparticle by ultrasound irradiation in-situ on the cotton fabric. Tragacanth gum (TG), a natural polysaccharide, was used as stabilizer. Our material exhibit larger particles with a very regular star- like morphology and additionally, there was now need to apply stabilizer to distribute them on cellulose fiber^22^.

**Figure 3:**
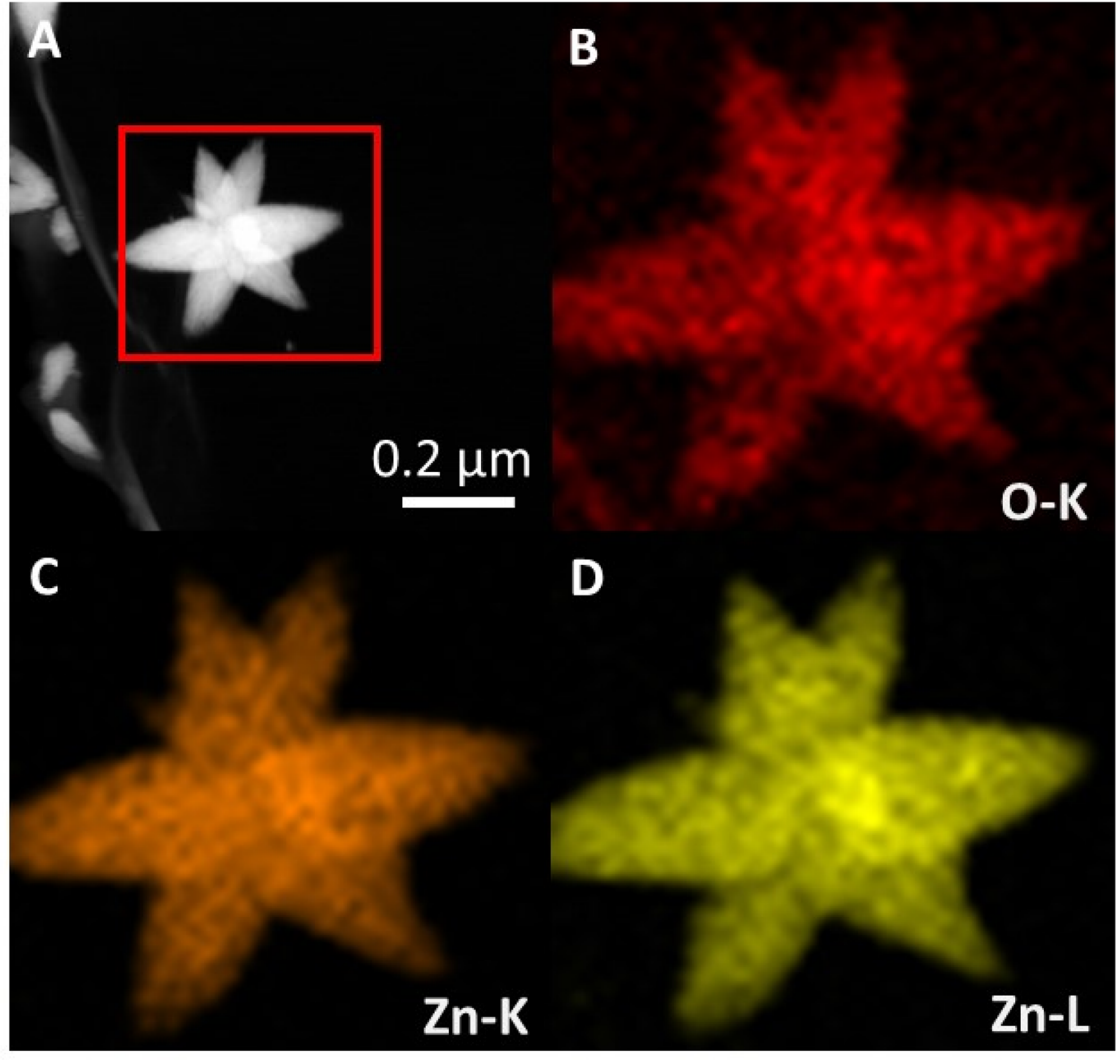
STEM images of the ZnO star (**A**) with corresponding elemental maps of characteristic X-ray: O K-line (**B**), Zn K-line (**C**) and Zn L-line (**D**). EDX maps were acquired from the region marked by a red square.

Figure 3 depicts the elemental analysis via EDX as TEM mode of single star-shaped structure. The results indicate that star-like structures are composed only of Zn and O elements.

**Figure 4:**
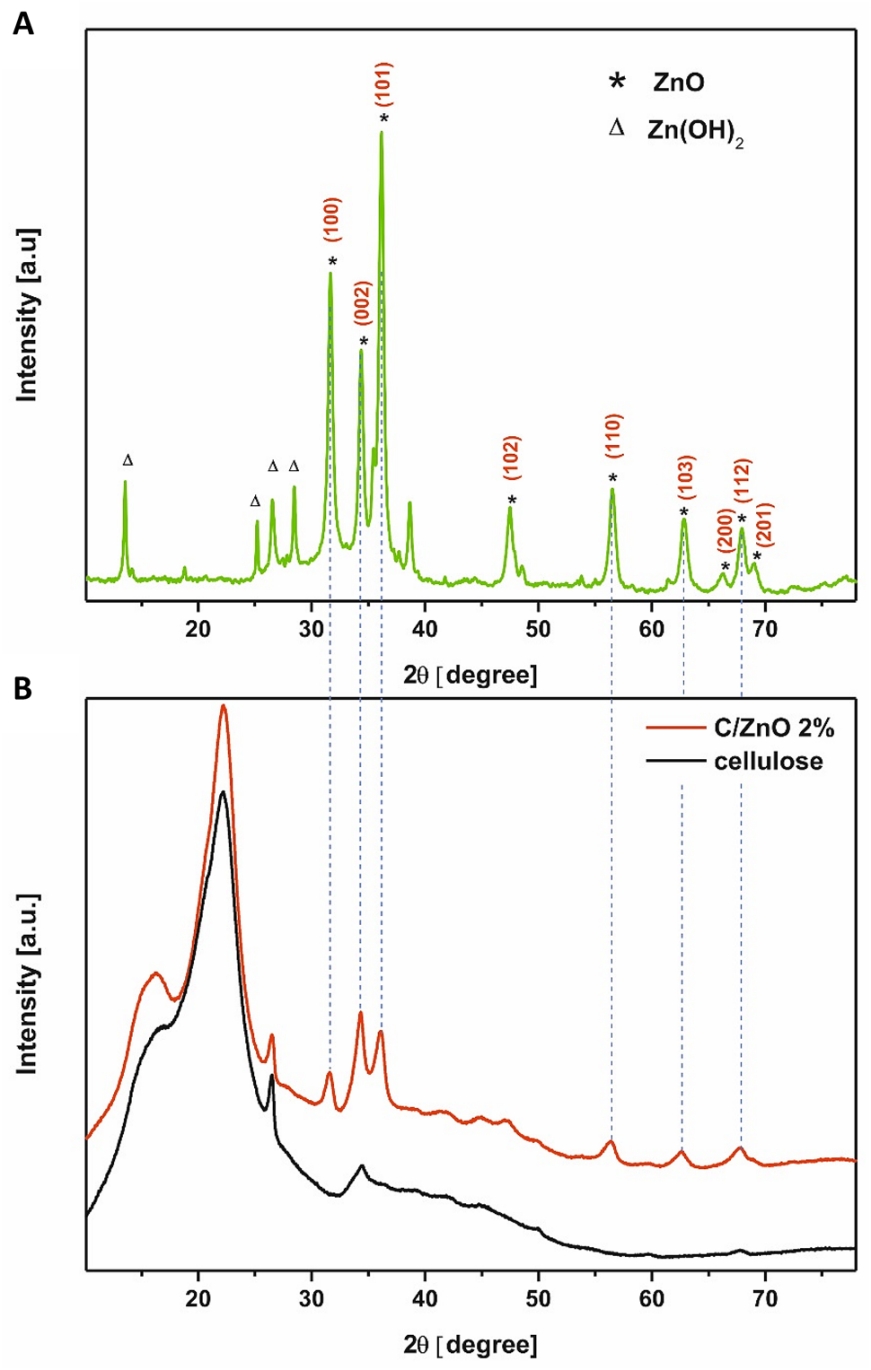
XRD patterns of post-combustion residue of functionalized cellulose fibers (**A**) and pure cellulose fibers (black curve) and cellulose fibers after the functionalization process (red curve) (**B**).

X-ray diffraction was used to reveal the crystal structure of the obtained materials. Figure 4A and B present XRD patterns of raw cellulose fibers and cellulose fibers functionalized with 2 wt% of ZnO. Two strong, broad peaks at 16.3 and 22.2° and one less intense and sharp reflection at 34.4° appear in both cellulose and C/ZnO. These signals are characteristic 110, 200, and 004 planes assigned to the typical cellulose-I structure^23,24^. A number of additional weak signals attributed to the presence of the nanofiller can be observed in the pattern of functionalized cellulose. Their low intensity is caused by small content of nanoparticles covering the surface of cellulose fibers, which makes hard to collect the diffraction data during the XRD examination. Thus, to unambiguously confirm crystalline structure of the nanofiller in the pads, the X-ray analysis of the residue collected after combustion of C/ZnO 2% sample was carried out. Combustion was conducted during thermo-gravimetric analysis. Figure 4A presents X-ray diffraction pattern of the sample recorded in the range of 10°–75°. Several sharp and intense Bragg reflections are observed at 31.65°, 34.36°, 36.16°, 47.49°, 56.51°, 62.91°, 67.88°, 69.03°, 72.59°, 77.17°. These reflections are well matched with the usually reported peak positions for bulk ZnO (in accordance with standard PDF card no. 01-079-0207) which confirms that synthesized powder is single crystalline and possesses a wurtzite hexagonal structure. The obtained XRD pattern displays few additional diffraction peaks in the range of 10°–30°. These comparatively weak signals are attributed to the presence of zinc hydroxide which is an intermediate product during the formation of ZnO in alkaline solution.

To fully understand how these structures were formed, the growth mechanism of ZnO nanoparticles will be presented. It begins with [Zn^+^] and [OH^−^] which in this case were provided by the Zn(CH_3_COO)_2_ and NaOH, respectively. No other nucleation-control medium or conditions were applied during the reaction process, therefore only concentration of [OH^−^] and [Zn^2+^] in the solution determined formation of the produced structures. Both Zinc and hydroxide ions form Zn(OH)_2_ (Eq. (1)), and when the saturation exceeds the critical value, nucleation occurs. Stable complex of [Zn(OH)_4_]^2−^ starts to form (Eq. (2)), which act as a growing unit of the ZnO nanostructures. When the concentration of [OH^−^] is greater than the [Zn^2+^] concentration, the intermediate product—[Zn(OH)_4_]^2^ dicomposes into ZnO and water molecules. Prolonged growing time and the low concentration of the [OH^−^] and [Zn^2+^] in the solution, resulted in the lack of interaction between clusters and ions, thus upon the final decomposition of [Zn(OH)_4_]^2−^ star-like ZnO were formed (Eq. (3))^25,26^.

1 $$ {\text{Zn}}^{2 + } + 2{\text{OH}}^{ - } \to {\text{Zn}}\left( {{\text{OH}}} \right)_{2} $$

2 $$ {\text{Zn}}\left( {{\text{OH}}} \right)_{2} + 2{\text{OH}}^{ - } \to \left[ {{\text{Zn}}\left( {{\text{OH}}} \right)_{4} } \right]^{2 - } $$

3 $$ \left[ {{\text{Zn}}\left( {{\text{OH}}} \right)_{4} } \right]^{2 - } \to {\text{ZnO}} + {\text{H}}_{2} {\text{O}} + 2{\text{OH}}^{ - } $$

**Figure 5:**
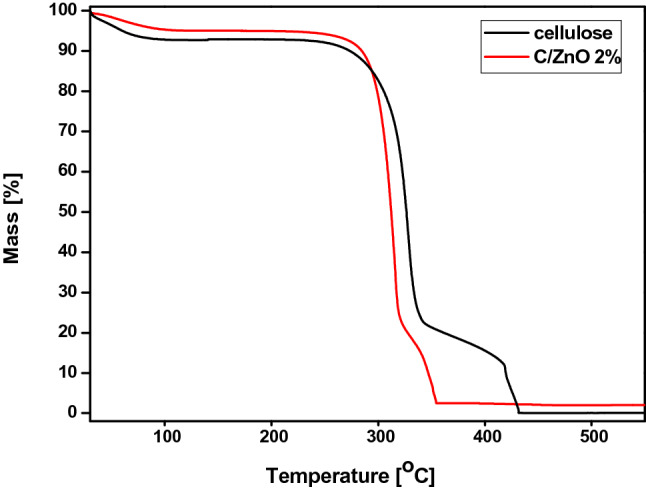
TGA of pure cellulose (black curve) and cellulose functionalized with 2 wt% of ZnO (red curve).

TGA is a tool used to monitor the composition as well as thermal stability of various materials via change in mass as a function of time under controlled conditions. Figure 5 shows the thermogravimetric analyses of pure cellulose and cellulose functionalized with 2 wt% of ZnO. For the pure cellulose sample the first weight loss of ~ 7 wt% at 80 °C is attributed to the evaporation of adsorbed water molecules. Another two thermal events at the temperature ranges of 200–340 °C and 340–430 °C that correspond to the thermal degradation of cellulose lead to the weight loss of 70 wt% and 23 wt%, respectively^27^. At the end of the degradation no residue can be detected which is consistent with the fact, that besides pristine cellulose, no fillers and chemicals were used during the preparation of the cellulose pad sheets. On the other hand cellulose functionalized with ZnO nanoparticles displayed weight loss of ~ 98% that consisted of thermal events at the temperature ranges from 165 to 320 °C and from 320 to 345 °C. Assuming that complete degradation of cellulose occurs by 500 °C, the cellulose functionalized with ZnO particles shows ~ 2 wt% of the residue. This value matches well with the calculated and introduced amount of the ZnO particles to get the composite. The TGA results also indicate that the thermal stability of the composite is reduced in comparison to pure cellulose as its thermal degradation curve shifted to lower temperature. This phenomenon can be ascribed to the high thermal conductivity of ZnO^28^. Due to direct interaction between cellulose and ZnO particles, the heat flows much easier within whole composite resulting in lower thermal decomposition temperature.

**Figure 6:**
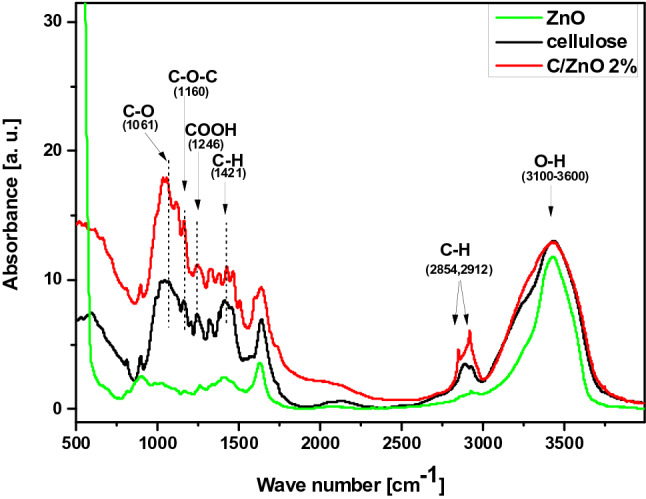
FTIR spectra of ZnO nanoparticles (green curve), cellulose fibers before (black curve) and after (red curve) the functionalization with ZnO particles.

**Figure 7:**
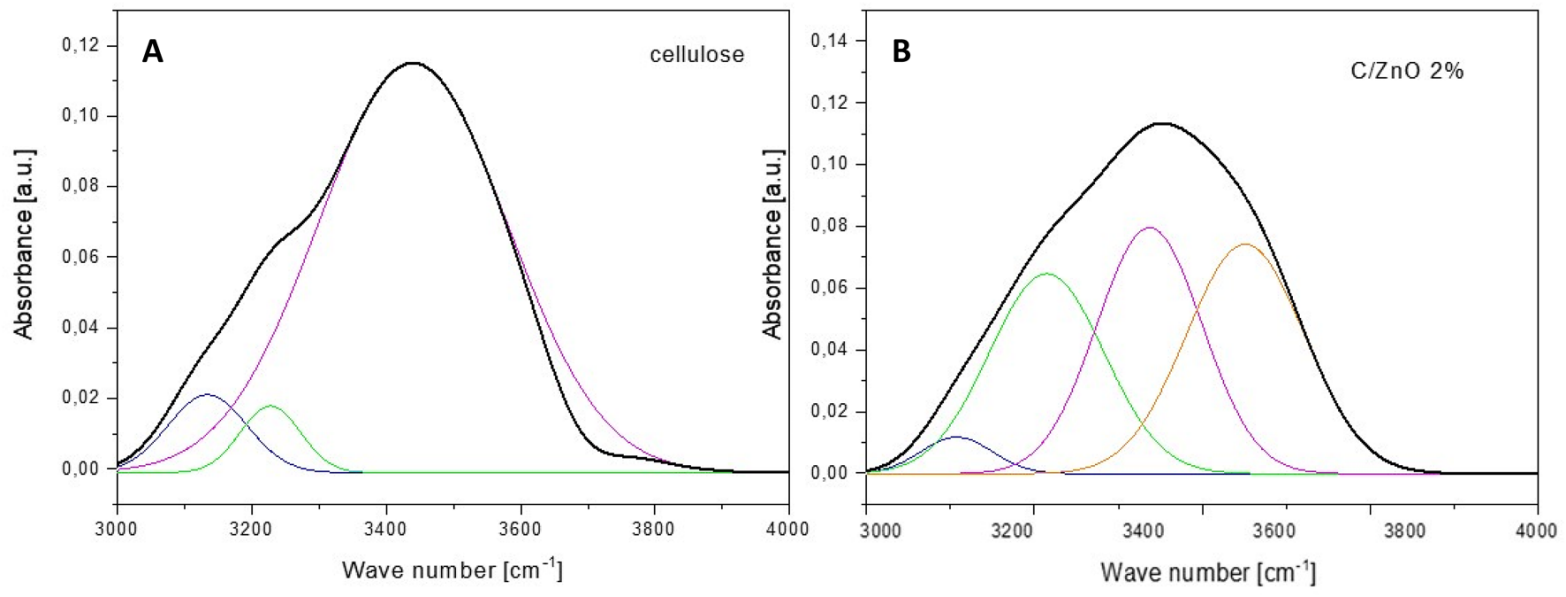
Deconvoluted spectra of the 3000–4000 cm^−1^ regions for raw cellulose fibers (**A**) and cellulose fibers functionalized with 2 wt% of ZnO particles (**B**).

In order to characterize the structural differences between cellulose fibers before and after functionalization, the samples were subjected to FT-IR tests and the results are presented in Fig. 6. Additionally, FT-IR spectra of pure ZnO nanoparticles synthesized via the same method but without the presence of cellulose is also provided. The infrared absorption spectra of raw cellulose fibers (black curve), cellulose fibers after the functionalization with 2 wt% of ZnO (red curve) and ZnO particles (green curve) were recorded in the 400–4000 cm^−1^ wave-number range. The characteristic absorption bands for cellulose are observed in two wave number regions: in the “fingerprint” region in 400–1650 cm^−1^ and the O–H and C–H stretching vibrations in 2700–3600 cm^−1^. The spectra of both cellulose samples in the “fingerprint” region are very complex. Although, no signal around 400–500 cm^−1^ assigned to the stretching mode of Zn–O bond recorded for obtained material can be observed in the C/ZnO spectrum (probably due to the very small amount of the nanofiller). Some obvious changes are detected in the FT-IR spectrum of the functionalized cellulose compared to the pure cellulose. Nevertheless, both samples display typical peaks that correspond to the different stretching vibrations of the cellulose functional groups, with the positions characteristic for pulp fibers. The broad band in 3100–3600 cm^−1^ region, related to the OH-stretching vibration, gives considerable information concerning the hydrogen bonds. This OH region always covers 3–4 sub-peaks which cannot be determined in the original data. Thus the deconvolution method was used to identify those peaks. The results of the deconvolution of FT-IR spectra of unfunctionalized and functionalized cellulose fibers in the range of 3000–4000 cm^−1^ are presented in Fig. 7A and B, respectively. The obtained results show clear changes in hydrogen-bonding pattern caused by the functionalization process. Change in the number of sub-peaks and their intensities can be observed which might indicate the rearrangement of the inter- and intra-chain H-bonds of the cellulose samples. However, it cannot be concluded clearly that during the functionalization, ZnO particles were bonded with cellulose fibers via hydrogen bonding. The infrared absorption spectra analysis also revealed significant changes in the spectral range of 2870–2950 cm^−1^ associated with C–H stretching of CH_2_ or CH_3_ in case of functionalized cellulose sample. It is clear also that two sharp peaks appeared at 2848 cm^−1^ and 2918 cm^−1^ in the spectra of C/ZnO can be assigned the formation of cellulose acetate. Dual functionalization of cellulose fibers was achieved using one-step precipitation method providing the cellulose acetate fibers functionalized with ZnO star-like objects.

**Figure 8:**
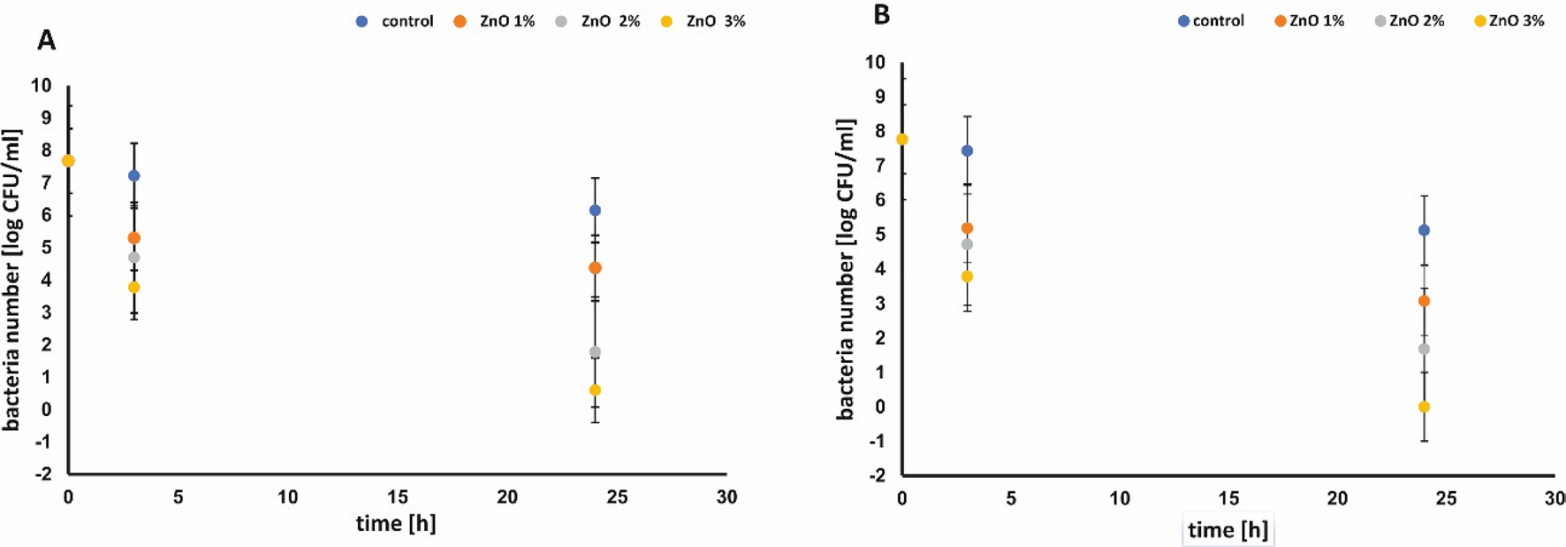
The antibacterial effects of functionalization of cellulose fibers with varied amount of ZnO *E. coli* (**A**), *S. epidermidis* (**B**).

**Figure 9:**
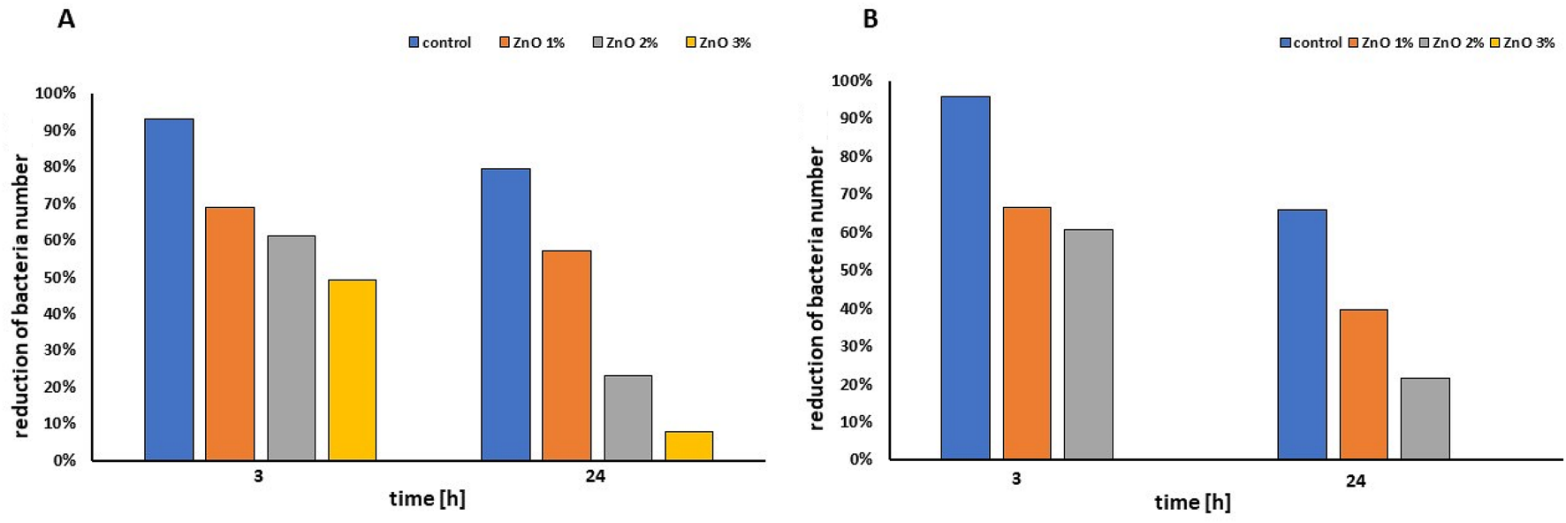
The percentage reduction of bacteria: *E. coli* (**A**), *S. epidermidis* (**B**) versus quantity of ZnO in functionalized paper and control.

After functionalization of the cellulose paper with different ZnO loading the antibacterial performance was investigated. The results of the antibacterial activity of ZnO NPs deposited on cellulose fibers and control samples against *E. coli* and *S. epidermidis* are shown in Fig. 8A and B, respectively. Figure 9A and B show the inhibition rate expressed as a percentage of bacterial reduction. After 3 h of Gram-negative *E*. *coli* incubation with the paper functionalized with 3% ZnO NPs a significant reduction (50.73%) of bacteria population was observed. Extension of contact time to 24 h caused further reduction in bacteria number (until 92.15%). In the same condition no Gram-positive bacteria *S. epidermidis* were noticed after 3 h (Fig. 9B). For the control samples, the reduction rate of *E*. *coli* and *S. epidermidis* population after 24 h was just 20.36% and 34.12%, respectively. As predicted, increasing zinc oxide content from 1 to 3 wt% deposited on the cellulose fiber resulted in improving antibacterial properties of papers against both model bacteria. This would suggest that the ZnO NPs are responsible for the antibacterial action. Obtained results were consistent with those reported by other authors. The antibacterial effect of ZnO particles toward Gram-negative and Gram-positive bacteria can vary. Higher susceptibility of Gram-positive bacteria could be related to differences in cell wall structure, degree of contact, different electrostatic affinity. This result was also reported by Vasile et al.^29^. Furthermore, as it was shown in the study of Xie et al.^30^ increase in the concentration of ZnO nanoparticles, its antibacterial effect also increases. Zinc oxide nanoparticles demonstrate good antibacterial properties against various bacteria strains even at low concentration. As it was reported by Ghayempour and Montazer^22^ the antimicrobial activity of tragacanth gum /ZnO nanoparticles synthesized on the cotton fabrics against *E. coli*, *S. aureu*s and *Candida albican*s still remains very good.

Antibacterial properties of ZnO–cellulose nanocomposite are very often determined using agar plate or agar disc-diffusion method that cannot be correlated with dynamic method applied in this study^31^. It is due to the fact that agar disk-diffusion technique do not allow to distinguish bactericidal and bacteriostatic effects or calculate amount of the antimicrobial agent diffused into the agar medium. Moreover, growth inhibition zones does not mean the bacterial death. For this reason better antibacterial effect against *E. coli* reported by Elemike et al. for cellulose ZnO/CNC materials could have resulted from better diffusion into agar media used for bacteria cultivation not for real antibacterial properties^18^. The discrepancy between observed antimicrobial performance for seemingly similar materials, has proved there is a urgent need to developed standardized method for screening and/or quantifying the antimicrobial effect of nanomaterials.

The ability to bacteria reduction was verified through ATP bioluminescence measurements. The results are shown in Table 2.

**Table 2:** ATP bioluminescence based on RLU**.**

Surface	RLU				
	Threshold values	After immersion in *E. coli* suspension	After 1.5 h incubation	After immersion in *S. epidermidis* suspension	After 1.5 h incubation
Raw cellulose fiber	15	191	114	264	142
Cellulose fibers functionalized with star-like zinc oxide nanoparticles	37	537	117	232	58

The higher RLU value after immersion in bacteria solution of the pads functionalized with 3 wt% ZnO confirm better water absorbency rate. The RLU percentage reduction after 1.5 h incubation soaked in bacterial solution of raw cellulose material was from 40.3 (for *E. coli*) to 46.2% (for *S. epidermidis*), while for the sample containing functionalized fibers from 78.2 (for *E. coli*) to 75.0 (for *S. epidermidis*). Significantly higher RLU reduction is in quite good agreement with results of antimicrobial test and has brought additional evidence of its antibacterial properties. It worth noting that Lumitester Smart Kikkoman System detects ATP from bacteria in addition to all other biological sources of ATP resulting from organic residues that can protect and provide a source of nutrients to microorganisms. However, decrease in RLU value correlates with lower bacterial number and determines cleaning properties of surface.

It is well known that zinc oxide is characterized by slight toxicity to human and animals. ZnO exhibited significant antimicrobial activity against broad spectrum of bacterial and fungal species^32–34^. For this reason it has been widely used as antibacterial agent as salts, coordinated compounds (complexes) or as the zinc oxide in skin creams, sunscreens, mouthwash, toothpaste and ointments^35^. It was proved that antimicrobial activity is influenced by ZnO concentration, the morphology of this compound and method of synthesis, particle size and presence of visible light in photocatalytic applications^36^. As it was shown in one of the latest review paper the highest potential antimicrobial effectiveness of nanoparticles against both *E. coli*, and *S. aureus* is observed at a nanoparticle size of about 100 nm as nanoparticles with small sizes (< 10 nm) are quite prone to aggregation. Nevertheless shape of ZnO nanoparticles is also crucial. Significantly bigger flower-shaped (up 3 µm) or thorn-or like ZnO nanoparticles demonstrated the antimicrobial activity against both gram-positive and gram-negative bacteria^37^. Additionally, Van Dong et al.^38^ and Penders et al.^39^ presented sharp edge and sharp vertex triangular silver nanoparticles showed better antimicrobial properties rounded nanoparticle. The antimicrobial efficacy of ZnO might be enhanced by the impact of star-shape nanoparticles promoting bacterial proliferation. Taking into account the above results, the star star-shaped structures, obtained in this study, are in the range of 1.304 µm ± 0.25 µm what perfectly fits in the model of ZnO antimicrobial action. A variety of antimicrobial mechanism of ZnO has been studied but there is no satisfactory explanation of that phenomena. It was proved that zinc oxide nanoparticles can lead to disruption of the cell membrane due to reaction with hydrogen ions forming molecules of H_2_O_2_. Moreover, it was reported that ZnO promoted the formation of other reactive oxidation species (ROS), responsible for oxidative damage of bacterial cells^37^. The main toxin resulting from exposure to hydrogen peroxide depends strongly on the surface area (on a higher surface area more oxygen species are produce)^40^. It is also possible that ZnO binding to proteins and DNA, trigger the processes of bacterial DNA amplification and expression in a wide range of genes^36,37^. As it was shown by Pasquet et al. the dissolution process of ZnO in various suspensions strongly influences its antimicrobial performance^32^. E.g. ZnO dispersion in pure water is very poor. Moreover, water is not a favorable medium for the growth of bacteria and Zn^2+^ ions exhibited lower antimicrobial activity than zinc complexes presented in more favorable for bacterial growth medium. It is expected that ingredients of liquid make-up remover such as : plant extracts, amino acids, glycerides, cause improvement of antimicrobial properties of obtained cellulose fibers used instead of classical cotton pads.

Over the past decade, the development of nano silver-containing materials, fabrics and cosmetics that exhibit antimicrobial and bactericidal properties has been applicated in may commercial applications^41–44^. On the other hand there are some alarming reports concerning adverse effect of AgNPs ubiquity exposure to human or emergence of silver-resistant bacteria^43^. In comparison to silver ZnO nanoparticles have several advantages: high antibacterial effectiveness at low concentrations against a wide range of bacterial and fungal strains at relatively low cost. More importantly, zinc compounds present limited absorption through human skin. It is well known that Zn^+2^ offers relatively few additive benefits due to antioxidant properties. It was confirmed that it can enhance the activity of antioxidant enzymes such as glutathione catalase (GSH) or superoxide dismutase (SOD), protect skin fibroblasts exposed to UVA and UVB radiation and decrease in cytotoxicity and lipid peroxidation and inhibits the formation of inflammatory mediators such as nitric oxide. Zinc compounds can be used as anti-ageing agents as well^35^.

To test the effect of the nanofiller on the mechanical properties of the material the control sample made of pristine cellulose fibers and the sample containing fibers functionalized with 2 wt% of ZnO were prepared. The grammage of both materials was ~ 60 g/m^2^. Table 3 summarizes the results on the examined mechanical properties. Tensile strength value of functionalized paper sheets increased by 38% over the non-functionalized paper. The same sample demonstrated 1.4 times greater value of tearing strength in comparison to the control cellulose fibers. Bursting strength value of the functionalized cellulose fibers increased significantly as well—it was approximately 2.2 times greater than that of the control sample. In comparison to the control sample, mechanical performance of the cellulose fabric contains such small amount of nanofiller as 2 wt% was significantly improved. These results are consistent with data from the literature in which it has been already proved that uniform distribution of nanofillers and its good adhesion to polymer matrix can significantly improve its mechanical properties^45^. In our case the formation of hydrogen bonding between cellulose fibers and ZnO nanoparticles with high surface to volume ratio could lead to the increase in its density. Free spaces between cellulose fibers could be successfully fulfilled which boosted the mechanical performance of functionalized cellulose paper sheets. Therefore, it is clearly seen that the functionalization of the cellulose-based fibers with star-like ZnO particles provides added value in the in the field of mechanically resistant and antibacterial technology.

**Table 3:** The effect of the nanofiller on the mechanical properties of cellulose fibers.

	Control	C/ZnO (2 wt%)
Tensile index [N m/g]	28.7 ± 1.3	46.3 ± 3.6
Tear index [mN m^2^/g]	4.7 ± 0.45	6.7 ± 0.54
Burst index [kPa m^2^/g]	1.2 ± 0.06	2.6 ± 0.08

**Figure 10:**
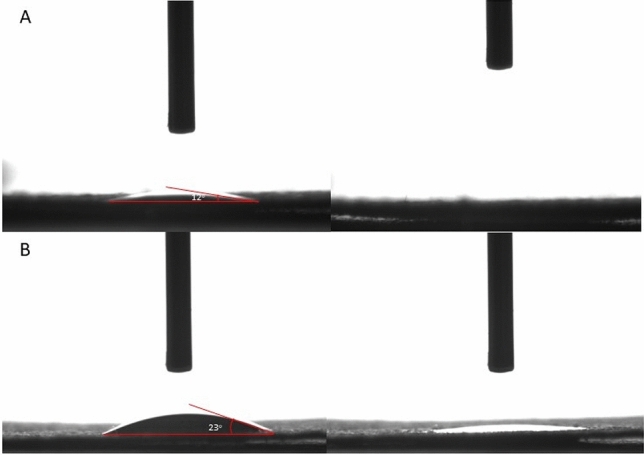
The profile of a water droplet on the surface of: cellulose fabric consisting of pure cellulose fibers (**A**), cellulose fibers functionalized with 3 wt% of ZnO structures (**B**).

Figure 10A, B presents the profile of a water droplet on the surface of cellulose fabric consisting of pure cellulose fibers. When first placed on the surface, the water droplet initially had a contact angle of ~ 12°. After less than a second the water droplet was fully absorbed into the cellulose fabric. Similar behavior was observed for the sample consisting of the cellulose fibers impregnated with 3 wt% of ZnO structures. The initial contact angle of the water droplet was ~ 23°. In less than one second the droplet spread out and the primary contact angle decreased to 0°. It is well known that pristine cellulose fibers possess hydrophilic features due to the abundance of OH functional groups. After the functionalization with ZnO particles the profile of water droplet has not shown significant changes which suggest that the impregnated fabric can still act as a water absorbing material.

High hydrophilicity and excellent adsorption ability of cellulose fibers functionalized with 3% ZnO can contribute to better skin hydration during daily care.

## Conclusions

A simple method was adopted to get durable functionality of cellulose and star-like ZnO particles based cosmetic pads with strong effect on antibacterial and mechanical performance and the same hydrophilic properties in comparison to pristine cellulose pads. The incorporation of additive into the cellulose matrix and its successful bonding with fibers was proven by several microscopic and spectroscopic methods. Regular star-shaped morphology of ZnO was developed in controlled precipitation method. Moreover, cellulose acetate was formed simultanouesly and the dual functionalization of cellulose fibers was obtained. Time dependent bactericidal activity of zinc oxide imprregnated cellulose composite was performed through colony forming method. Paper pads prepared from modified cellulose fibers with different loadings of nanofiller presented strong antibacterial performance against both Gram-negative (*E. coli*) and Gram-positive (*S. epidermidis*) bacteria in a comparision with raw cellulose paper sheets. Incorporation of ZnO nanoparticles into cellulose matrix decreased the viable cell count that was also concentration dependent. 3 h of *E*. *coli* incubation with the paper functionalized with 3 wt% of ZnO NPs caused reduction of bacteria population by 50.73%. After further extension of contact time up to 24 h the reduction in bacteria number by 92.15% was observed. In the same condition no gram positive bacteria *S. epidermidis* were noticed after only 3 h of the incubation. Additional disinfection ability of obtained materials was confirmed by luminescence test. Significant improvement of the mechanical properties was observed after the incorporation of only 2 wt% of nanofiller which is an added value of the obtained antibacterial paper. Thus, such kind of biopolymer nanocomposite, characterized by high hydrophilicity could be potentially used in a cosmetic industry for a variety of applications where bactericidal features are required, in particular for fabrication of new type of cellulose pads for makeup removal.

## References

[1] Thiruvengadam M, Rajakumar G, Chung IM (2018). Nanotechnology: Current uses and future applications in the food industry. 3 Biotech.

[2] Sharma C, Dhiman R, Rokana N, Panwar H (2017). Nanotechnology: An untapped resource for food packaging. Front. Microbiol..

[3] Espitia PJP, Soares NFF, Coimbra JSR, Andrade NJ, Cruz RS, Medreiros EAA (2012). Zinc oxide nanoparticles: Synthesis, antimicrobial activity and food packaging applications. Food Bioprocess. Technol..

[4] Roduner E (2006). Size matters: Why nanomaterials are different. Chem. Soc. Rev..

[5] Lee J, Mahendra S, Alvarez PJJ (2010). Nanomaterials in the construction industry: A review of their applications and environmental health and safety considerations. ACS Nano.

[6] Azam A, Ahmed AS, Oves M, Khan MS, Habib SS, Memic A (2012). Antimicrobial activity of metal oxide nanoparticles against Gram-positive and Gram-negative bacteria: A comparative study. Int. J. Nanomed..

[7] Fu G, Vary PS, Lin CT (2005). Anatase TiO_2_ nanocomposites for antimicrobial coatings. J. Phys. Chem. B.

[8] Ling CA, Shahrom M, Bakhori SKM, Sirelkhatim A, Mohamad D, Hasan H, Seeni A, Rahman RA (2014). Antibacterial responses of zinc oxide structures against *Staphylococcus aureus*, *Pseudomonas aeruginosa* and *Streptococcus pyogenes*. Ceram. Int..

[9] Tang ZX, Lv BF (2014). MgO nanoparticles as antibacterial agent: Preparation and activity. Braz. J. Chem. Eng..

[10] Mahapatra O, Bhagat M, Gopalakrishnan C, Arunachalam KD (2008). Ultrafine dispersed CuO nanoparticles and their antibacterial activity. J. Exp. Nanosci..

[11] Yetisen AK, Qu H, Manbachi A, Butt H, Dokmeci MR, Hinestroza JP, Skorobogatiy M, Khademhosseini A, Yun SH (2016). Nanotechnology in textiles. ACS Nano.

[12] Karlsson HL, Cronholm P, Hedberg Y, Tornberg M, De Battice L, Svedhem S, Wallinder IO (2013). Cell membrane damage and protein interaction induced by copper containing nanoparticles—Importance of the metal release process. Toxicology.

[13] Markowska-Szczupak A, Endo-Kimura M, Paszkiewicz O, Kowalska E (2020). Are titania photocatalysts and zitanium implants safe? Review on the toxicity of titanium compounds. Nanomaterials (Basel).

[14] Xiao Y, Liu Y, Kang S, Wang K, Xu H (2020). Development and evaluation of soy protein isolate-based antibacterial nanocomposite films containing cellulose nanocrystals and zinc oxide nanoparticles. Food Hydrocoll..

[15] Pillai AM, Sivasankarapillai VS, Rahdar A, Joseph J, Sadeghfar F, Anuf AR, Rajesh K, Kyzas GZ (2020). Green synthesis and characterization of zinc oxide nanoparticles with antibacterial and antifungal activity. J. Mol. Struct..

[16] Raghupathi KR, Koodali RT, Manna AC (2011). Size-dependent bacterial growth inhibition and mechanism of antibacterial activity of zinc oxide nanoparticles. Langmuir.

[17] Azeredo HMC (2013). Antimicrobial nanostructures in food packaging. Trends Food Sci. Technol..

[18] Elemike E, Onwudiwe D, Mbonu J (2021). Facile synthesis of cellulose–ZnO–hybrid nanocomposite using *Hibiscus rosa-sinensis* leaf extract and their antibacterial activities. Appl. Nanosci..

[19] Gao S, Gao R (2017). Antibacterial cellulose composite membranes prepared in ionic liquid via phase inversion method. Chem. Res. Chin. Univ..

[20] Ali A, Ambreen S, Maqbool Q, Naz S, Shams MF, Ahmad M, Phull AR, Zia M (2016). Zinc impregnated cellulose nanocomposites: Synthesis, characterization and applications. J. Phys. Chem. Solids.

[21] Zemjic L, Valh J, Kreze T (2017). Preparation of antibacterial paper sheets using chitosan. Cell. Chem. Technol..

[22] Ghayempour S, Montazer M (2017). Ultrasound irradiation based in-situ synthesis of star-like Tragacanth gum/zinc oxide nanoparticles on cotton fabric. Ultrason. Sonochem..

[23] Kumar A, Negi JS, Choudhary V, Bhardwaj NK (2014). Characterization of cellulose nanocrystals produced by acid-hydrolysis from sugarcane bagasse as agro-waste. J. Mater. Phys. Chem..

[24] Zhao SW, Guo CR, Hu YZ, Guo YR, Pan QJ (2018). The preparation and antibacterial activity of cellulose/ZnO composite: A review. Open Chem..

[25] Fan J, Li T, Heng H (2014). Hydrothermal growth and optical properties of ZnO nanoflowers. Mater. Res. Express.

[26] Pérez-Hernández R (2010). Hydrogen production by steam reforming of methanol over a Ag/ZnO one dimensional catalyst. Adv. Mater. Res..

[27] Dahiya JB, Rana S (2004). Thermal degradation and morphological studies on cotton cellulose modified with various arylphosphorodichloridites. Polym. Int..

[28] Zhao SW, Zheng M, Zou XH, Guo Y, Pan QJ (2017). Self-assembly of hierarchically structured cellulose@ZnO composite in solid–liquid homogeneous phase: Synthesis, DFT calculations, and enhanced antibacterial activities. ACS Sustain. Chem. Eng..

[29] Vasile C, Râpă M, Ștefan M, Stan M, Macavei S, Darie-Niță RN, Barbu-Tudoran L, Vodnar DC, Popa EE, Ștefan R, Borodi G, Brebu M (2017). New PLA/ZnO:Cu/Ag bionanocomposites for food packaging. XPRESS Polym. Lett..

[30] Xie Y, He Y, Irwin PL, Jin T, Shi X (2011). Antibacterial activity and mechanism of action of zinc oxide nanoparticles against *Campylobacter jejuni*. Appl. Environ. Microbiol..

[31] Sirelkhatim, A. *et al.* Review on zinc oxide nanoparticles: Antibacterial activity and toxicity mechanism. *Nanomicro Lett.* **7**(3):219-242. 10.1007/s40820-015-0040-x (2015). 10.1007/s40820-015-0040-x PMC6223899 30464967

[32] Pasquet J, Chevalier Y, Couval E, Bouvier D, Noizet G, Morlière C, Bolzinger MA (2014). Antimicrobial activity of zinc oxide particles on five micro-organisms of the challenge tests related to their physicochemical properties. Int. J. Pharm..

[33] Jones N, Ray B, Ranjit KT, Manna AC (2008). Antibacterial activity of ZnO nanoparticle suspensions on a broad spectrum of microorganisms. FEMS Microbiol. Lett..

[34] Zhang L, Jiang Y, Ding Y, Povey M, York D (2007). Investigation into the antibacterial behavior of suspensions of ZnO nanoparticles (ZnO nanofluids). J. Nanopart. Res..

[35] Abendrot M, Kalinowska-Lis U (2018). Zinc-containing compounds for personal care applications. Int. J. Cosmet. Sci..

[36] Duffy LL, Osmond-McLeod MJ, Judy J, King T (2018). Investigation into the antibacterial activity of silver, zinc oxide and copper oxide nanoparticles against poultry-relevant isolates of Salmonella and Campylobacter. Food Control.

[37] Gudkov SV, Burmistrov DE, Serov DA, Rebezov MB, Semenova AA, Lisitsyn AB (2021). A mini review of antibacterial properties of ZnO nanoparticles. Front. Phys..

[38] Van Dong P, Ha CH, Binh LT, Kasbohm J (2012). Chemical synthesis and antibacterial activity of novel-shaped silver nanoparticles. Int. Nano Lett..

[39] Penders J, Stolzoff M, Hickey DJ, Andersson M, Webster TJ (2017). Shape-dependent antibacterial effects of non-cytotoxic gold nanoparticles. Int. J. Nanomed..

[40] Gunalan S, Sivaraj R, Rajendran V (2012). Green synthesized ZnO nanoparticles against bacterial and fungal pathogens. Prog. Nat. Sci..

[41] Ma J, Zhu W, Tian Y, Wang Z (2016). Preparation of zinc oxide-starch nanocomposite and its application on coating. Nanoscale Res. Lett..

[42] Yunsov KHE, Mullajonova SV, Sarymsakov AA, Jalilov JZ, Turakulov FM, Rashidova SSH, Lutfullin R (2019). Antibacterial effect of cotton fabric treated with silver nanoparticles of different sizes and shapes. Int. J. Nanomater. Nanotechnol. Nanomed..

[43] Rezvani E, Rafferty A, McGuinness C, Kennedy J (2019). Adverse effects of nanosilver on human health and the environment. Acta Biomater..

[44] El-Nahhal IM, Salem J, Anbar R, Kodeh FS, Elmanama A (2020). Preparation and antimicrobial activity of ZnO–NPs coated cotton/starch and their functionalized ZnO–Ag/cotton and Zn(II) curcumin/cotton materials. Sci. Rep..

[45] Gao N, Hou G, Liu J, Shen J, Gao Y, Lyulin A, Zhang A (2019). Tailoring the mechanical properties of polymer nanocomposites via interfacial engineering. Phys. Chem. Chem. Phys..

